# The bioinformatics and experimental analysis of the novel roles of virus infection-associated gene CDC20 for prognosis and immune infiltration in hepatocellular carcinoma

**DOI:** 10.18632/aging.204093

**Published:** 2022-05-27

**Authors:** Juanni Li, Xiaofang Zhang, Lei Yao, Kuan Hu

**Affiliations:** 1 Department of Pathology, Xiangya Hospital, Central South University, Changsha 410008, Hunan, China; 2 National Clinical Research Center for Geriatric Disorders, Xiangya Hospital, Central South University, Changsha 410008, Hunan, China; 3 Departments of Burn and Plastic, Ningxiang People’s Hospital, Hunan University of Chinese Medicine, Changsha 410600, Hunan, China; 4 Department of Hepatobiliary Surgery, Xiangya Hospital, Central South University, Changsha 410008, Hunan, China

**Keywords:** HTLV-1, CDC20, hepatocellular carcinoma, prognosis, immune infiltration

## Abstract

Infection virus including HBV and HCV has been well recognized as a major cause inducing hepatocellular carcinoma (HCC). However, molecular investigations into the HTLV-1 (Human T-lymphotropic virus type-1) and HCC have been rare. In this study, we integrated several public datasets of HCC patients and filtered seven genes including CDC20 as the HTLV-1 infection-related genes which were differentially expressed in HCC. CDC20 was chosen for further investigation based on its promising prognostic power. The expression profiles, prognostic assessment, association with clinicopathologic characteristics, prediction of correlated signal pathways, and the immune-modulating function of CDC20 were assessed. We found that CDC20 expression was significantly increased in hepatocellular carcinoma tissues and cell lines, and was correlated with histologic grade, pathologic stage, tumor status, and patient age. CDC20 exhibited prognostic value on overall survival and disease specific survival and was an independent prognostic factor. It was primarily involved in several signal pathways, especially the omega-hydroxylase P450 and epoxygenase P450 signal pathways. Moreover, CDC20 expression showed significant positive associations with the levels of several immune cells such as T helper 2 cells and follicular helper T cells, immunostimulators including TNFRSF18 and MICB, immunoinhibitors including KDR and PDCD1LG2, chemokines including XCL1 and CCL26, and chemokine receptors including CCR10 and CXCR3. This study for the first time delineated the correlation of CDC20 with HTLV-1 infection-associated HCC. The disorder of expression and function of CDC20 makes it a probable biomarker for better etiological classification, prognostic prediction, and precision medicine.

## INTRODUCTION

Hepatocellular carcinoma (HCC) is the most common type of liver cancer, the incidence rate of which ranks sixth worldwide. HCC is characterized by its concealment during initiation and high heterogeneity resulting from multiple risk factors of oncogenesis [1]. Among a variety of risk factors, virus infection including HBV and HCV has been well recognized as a major cause that induces the HCC through a series of complicated mechanisms between virus and host hepatocytes [2]. There were numerous studies aiming at unveiling the role of viruses like HBV and HCV in HCC these decades and consequently tremendous development has been made. However, to our knowledge, the investigations about the other viruses and HCC has been rare.

HTLV-1 (Human T-lymphotropic virus type-1) was first identified as a human retrovirus and caused infection of lymphocytes through the hematogenous spread, sex, and mother-to-fetus transmission, eventually resulting in adult T-cell leukemia/lymphoma (ATL) [3]. Since then, successively reports revealed the involvement of HTLV-1 in different human diseases ranging from myelopathy to rheumatic diseases, polymyositis, and uveitis [4–6]. Recently, the association between HTVL-1 and HCC has been preliminary illustrated, getting the conclusion that HTLV-1 might promote the tumor progression in HCC patients with an HCV background. Based on this intriguing finding, further molecular biological investigations and mechanism research on HTVL-1 and HCC are still lacking.

Hence in this present study, we integrated several public datasets of HCC patients and filtered the HTLV-1 infection-related genes which were differentially expressed in tumors. The most differential expression gene CDC20 was picked out to have significant effects on the development and prognosis of HCC among the seven candidate genes via systematical analyses in terms of expression profiles, prognostic assessment, association with clinicopathologic characteristics, prediction of correlated signal pathways, and the immune modulating function. CDC20 was known to serve as the key activator of Anaphase Promoting Complex (APC), a famous E3 ubiquitin ligase enzyme that participated in the cell cycle progression and determined the targeting substrate for further destruction [7]. Moreover, CDC20 was also reported to be an oncogene in several kinds of cancer including HCC [8]. However, the role of CDC20 as an HTLV-1 infection-related gene in HCC has not yet been illustrated. We believed this study may supply a novel perspective for a better understanding of the virus infection and their related genes in HCC, and help classify HCC patients with HBC/HCV into subtypes with more precise and specific regimens.

## RESULTS

### Identification of differentially expressed genes

To screen the differentially expressed genes (DEGs) in hepatocellular carcinoma, we compared HCC tumor tissues and normal liver tissues in four publicly available datasets including GSE101685, GSE112790, GSE45267, and GSE84402. Using the filtering criteria of *p*-value < 0.05 and |log2 Fold Change|>2, 165 genes in GSE101685, 121 genes in GSE112790, 78 genes in GSE45267, and 208 genes in GSE84402 were significantly up-regulated in HCC tumor tissues. In addition, we also identified 351, 206, 232, and 308 down-regulated genes in GSE101685, GSE112790, GSE45267 and GSE84402, respectively (Supplementary Table 1). Next, using the Venn diagram, 54 co-DEGs were identified to be significantly up-regulated, and 125 co-DEGs were found to be down-regulated in these four datasets. These co-DEGs were hypothesized to have a significant impact on the tumorigenesis and progression of hepatocellular carcinoma.

**Figure 1:**
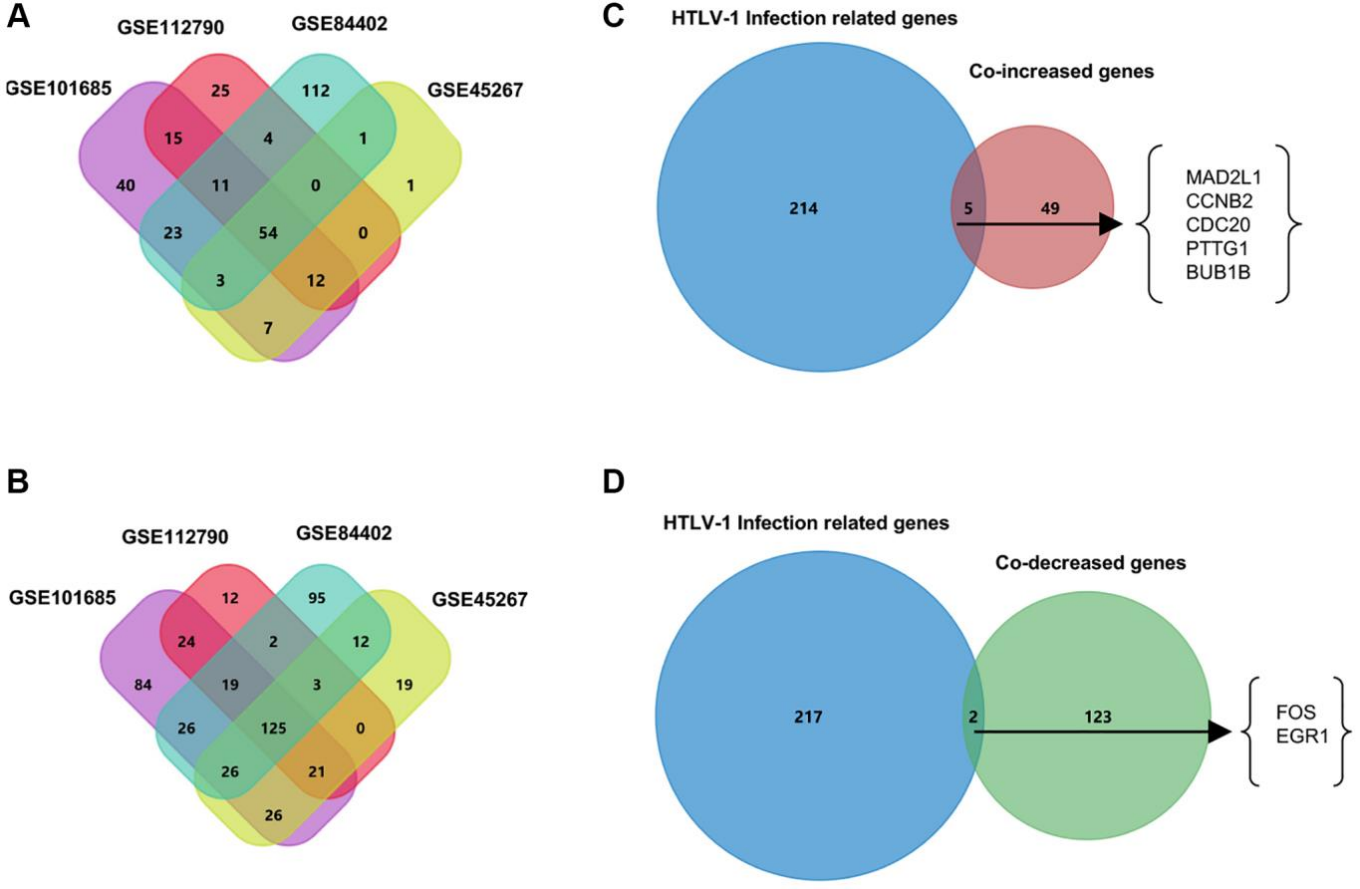
**Venn diagrams to filter HTLV-1 infection-associated genes from HCC datasets.** (**A**) The 54 co-increased expression genes were filtered from four public datasets GSE101685, GSE112790, GSE45267, and GSE84402. (**B**) The 125 co-decreased expression genes were filtered from four public datasets. (**C**) Five genes were identified as the up-regulated overlapping genes between HTLV-1 infection-related genes and the 54 co-DEGs. (**D**) FOS and EGR1 were identified as the two down-regulated overlapping genes between HTLV-1 infection-related genes and the 125 co-DEGs.

Virus infections take a significant part in the tumorigenesis and development of hepatocellular carcinoma [9–11]. Next, we investigate the possible roles of HTLV-1 infections on the progression of HCC. The HTLV-1 infection-related gene datasets acquired from the MalaCards database were shown in Supplementary Table 2. We applied the Venn diagram to identify the overlapping genes between HTLV-1 infection-related genes and the aforementioned co-DEGs. As shown in Figure 1, five HTLV-1 infection-related genes, MAD2L1, CCNB2, CDC20, PTTG1 and BUB1B were highly expressed, and two genes, FOS and EGR1, were lowly-expressed (Figure 1). These seven HTLV-1 infection-related genes were presumed to play critical roles on virus infection-associated development of hepatocellular carcinoma.

### CDC20 shows the greatest prognostic value in HCC

**Figure 2:**
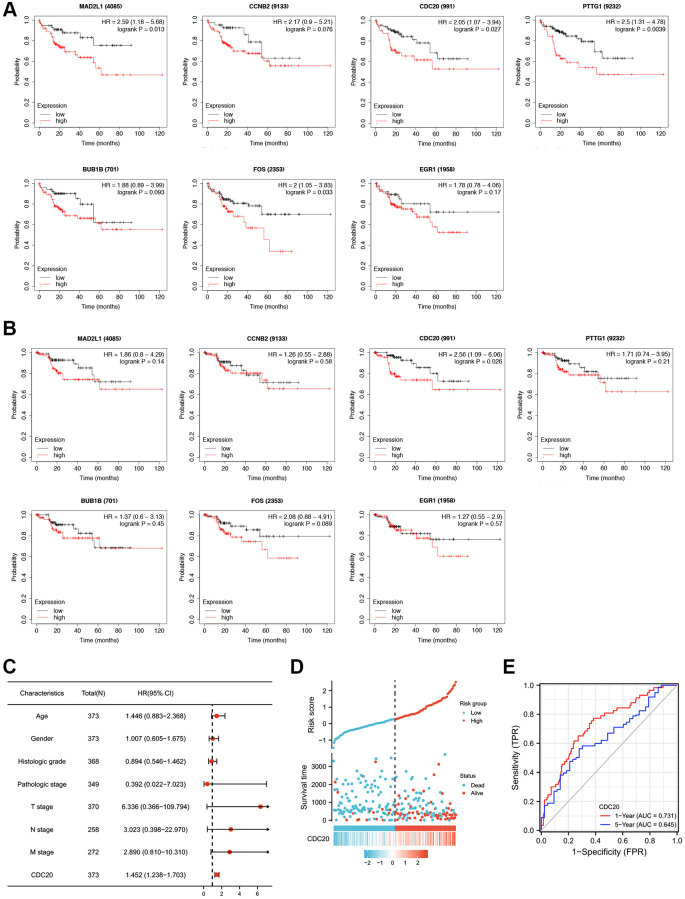
**Prognostic values of seven HTLV-1 infection-associated genes including CDC20 in HCC.** (**A** and **B**) Kaplan-Meier analysis of Overall Survival (OS) (**A**) and Disease Specific Survival (DSS) (**B**) in patients with differential expression of each HTLV-1 associated gene. (**C**) The forest figure revealed the multivariate Cox analysis of CDC20 expression and other clinicopathological variables. (**D**) CDC20 expression distribution and survival status. (**E**) ROC curves of CDC20.

HTLV-1 coinfection has been reported to have an influence on the development of HCC in patients with hepatitis virus infection [12]. Here, we used the Kaplan–Meier plotter to explore the prognostic values of mRNA expression of the aforementioned seven HTLV-1 infection-related genes in HCC patients with hepatitis virus infection. As shown in Figure 2A and Supplementary Figure 1A, higher mRNA expression levels of MAD2L1, CDC20, PTTG1 and FOS were significantly associated with shorter overall survival (OS). Meanwhile, higher mRNA expression levels of CDC20 was obviously associated with shorter disease specific survival (DSS) (Figure 2B, Supplementary Figure 1B). Based on the above results, CDC20, as the only gene that exhibited prognostic value on OS and DSS at the same time, was presumed as a more potential prognostic target and chose for further investigation.

Furthermore, we conducted a multivariate analysis to assess the correlation between overall survival and multivariable characteristics, and the results showed that CDC20 expression was an independent factor for patient prognosis (Figure 2C, Supplementary Table 3). Figure 2D exhibited the distribution of CDC20 expression, the survival time of patients with HCC, and risk score profiles. Moreover, the ROC curve indicated that CDC20 expression possesses promising prognostic power. The AUC of CDC20 expression was 0.731 and 0.645 for predicting 1-year and 5-year overall survival, respectively (Figure 2E). These results suggest that CDC20 is a promising prognostic biomarker in patients with HCC.

### CDC20 expression and its association with clinicopathological features in HCC

**Figure 3:**
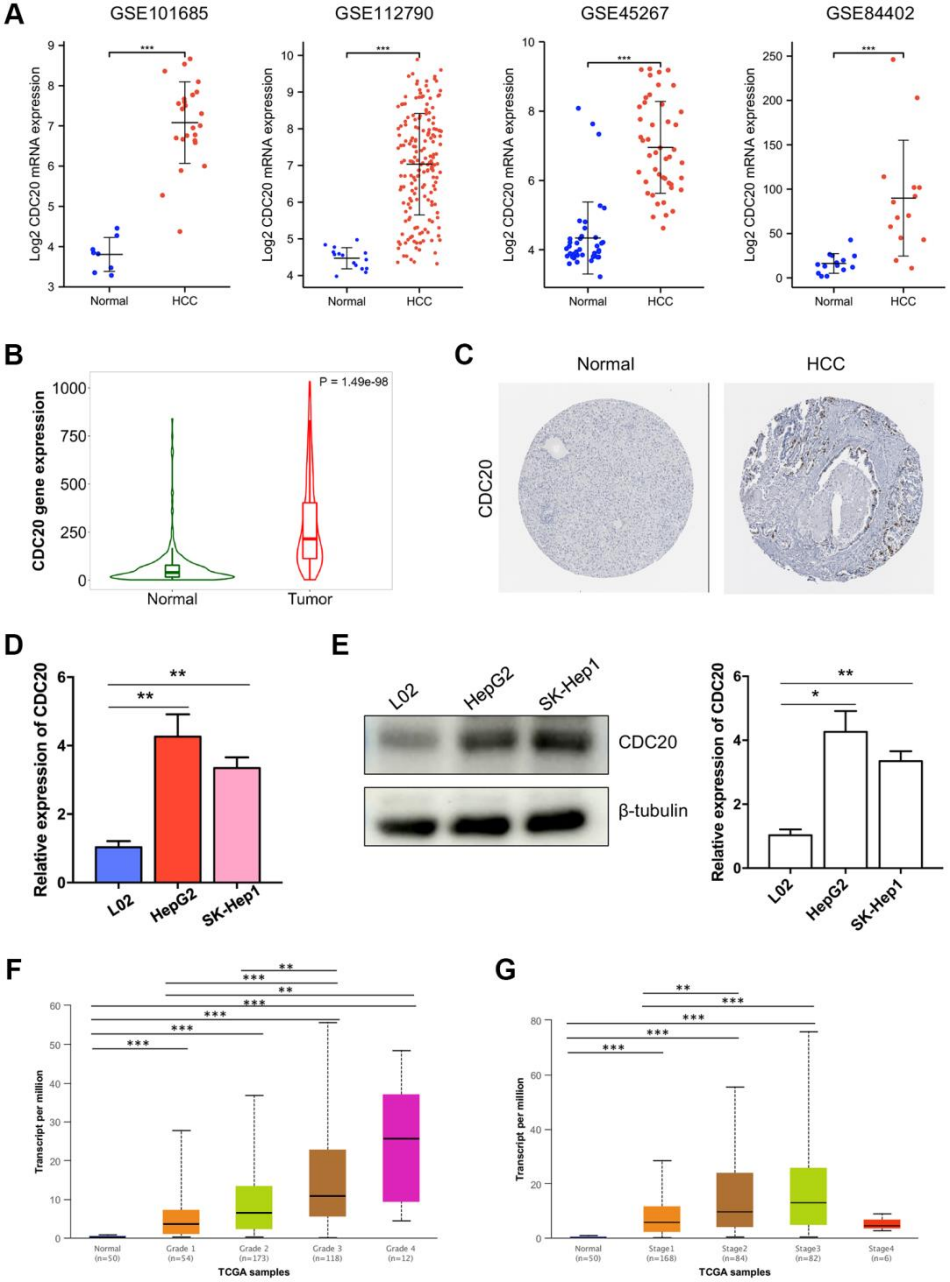
**Associations between CDC20 and clinicopathological parameters in HCC.** (**A**) Compared to normal liver tissues, the mRNA expression of CDC20 was increased in hepatocellular carcinoma tissues from four public HCC datasets. (**B**) TNM plot also showed the increased mRNA expression of CDC20 in hepatocellular carcinoma tissues. (**C**) Compared with the random normal liver tissues, the protein expression of CDC20 was increased in HCC tissues (the Human Protein Atlas, THPA). (**D** and **E**) qPCR and western blot showed that compared with the normal hepatocyte cell line L02, CDC20 expression was increased in hepatocellular carcinoma cell lines HepG2 and SK-Hep1. (**F** and **G**) The correlation of CDC20 with the tumor grades and cancer stages in patients with HCC.

The expression profiles of CDC20 were further confirmed using several databases and *in vitro* experiments. First, four aforementioned datasets, GSE101685, GSE112790, GSE45267, and GSE84402, exhibited that the transcriptional level of CDC20 was significantly up-regulated in HCC tumor tissues than in normal liver tissues (Figure 3A). Then, the TNM plot and THPA database showed that the mRNA and protein expression levels of CDC20 were significantly higher in HCC tissues (Figure 3B and 3C). Furthermore, the up- regulation of CDC20 transcriptional and protein expression was confirmed in hepatocellular carcinoma cells HepG2 and SK-Hep1 compared with the normal liver cell L02 through qPCR and Western Blot. These results showed that CDC20 expression was significantly increased in hepatocellular carcinoma tissues and cell lines (Figure 3D and 3E).

Moreover, the relationship between the CDC20 expression and clinicopathological features of HCC was further investigated. According to the UALCAN database, the mRNA expression of CDC20 was identified to be significantly correlated with the tumor grade and the cancer stage of HCC. The results showed that the higher tumor grade, the higher CDC20 expression in HCC. Patients who had higher cancer stages (stage 1–3) tended to have higher CDC20 expression, while patients with stage 4 showed low expression of CDC20, which may be due to the small sample size and other factors (Figure 3F and 3G). In addition, we also used the XianTao tool to analyze the clinical data of HCC patients. Consist with the above results, CDC20 expression was found to be significantly correlated with histologic grade and pathologic stage. And beyond that, CDC20 expression was also assessed to be significantly correlated with tumor status and patient age (Table 1). These findings indicated that patients with higher CDC20 expression were more prone to having hepatocellular carcinoma that was more advanced in grade, stage, and status.

**Table 1:** Association between the mRNA expression level of CDC20 and clinicopathologic characteristics using logistic regression.

**Characteristics**	**Total (*N*)**	**Odds Ratio (OR)**	***P* value**
Age (>60 vs. ≤60)	373	0.629 (0.417–0.946)	0.026
Gender (Male vs. Female)	374	0.764 (0.494–1.179)	0.225
Histologic grade (G3&G4 vs. G1&G2)	369	2.990 (1.930–4.685)	<0.001
Pathologic stage (III&IV vs. I&II)	350	1.805 (1.112–2.962)	0.018
T stage (T3&T4 vs. T1&T2)	371	1.803 (1.121–2.929)	0.016
N stage (N1 vs. N0)	258	0.954 (0.113–8.049)	0.963
M stage (M1 vs. M0)	272	0.305 (0.015–2.414)	0.306

### The generation of CDC20-associated network and the functional enrichment analysis of CDC20 in HCC

**Figure 4:**
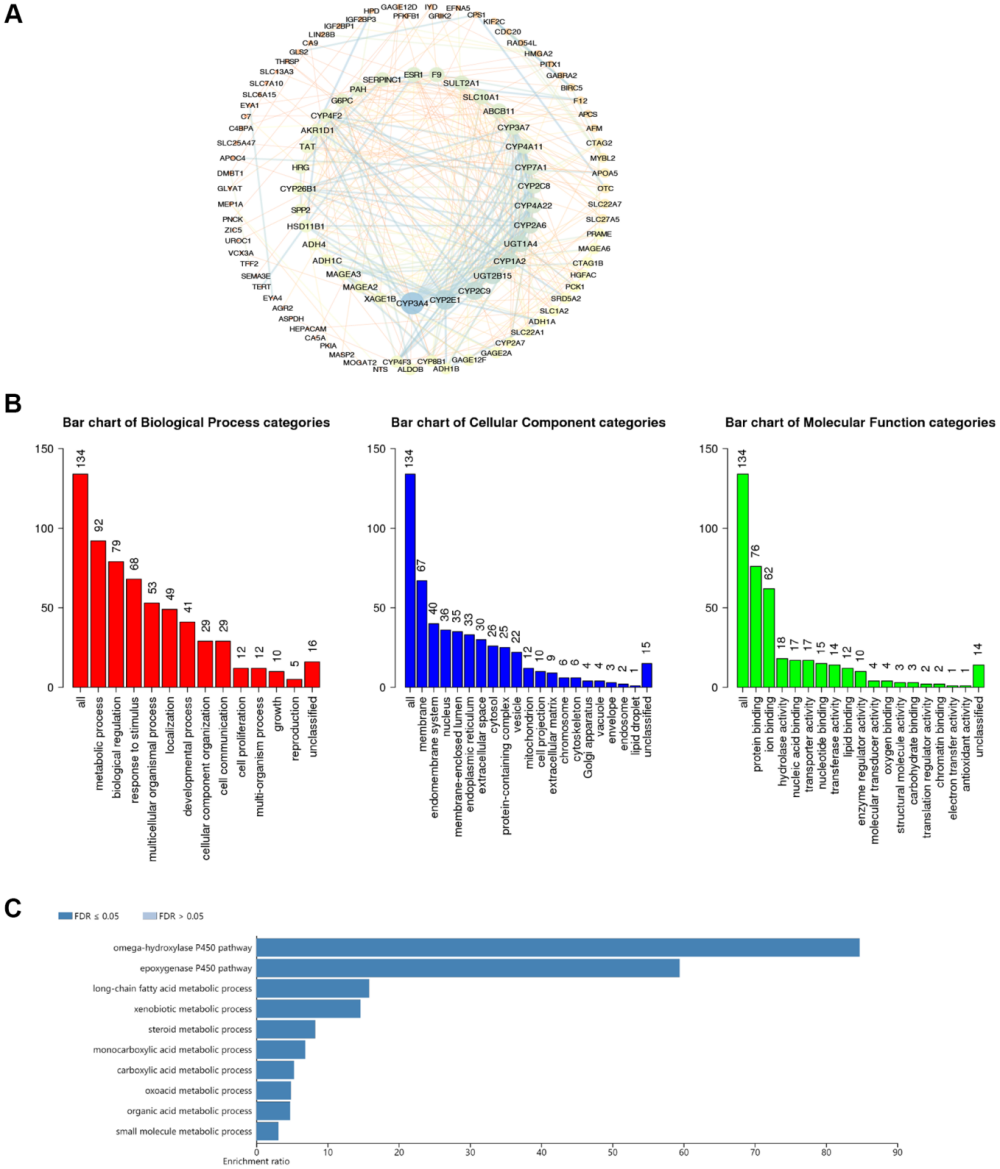
**The functional enrichment analysis of CDC20-associated genes in HCC.** (**A**) PPI network of CDC20-associated genes (cBioPortal and Cytoscape). The colors of edges and nodes: yellow color means low value, blue color means high value. (**B**) Gene Ontology (GO) analysis of CDC20-associated genes (WebGestalt). (**C**) Kyoto Encyclopedia of Genes and Genome (KEGG) pathway analysis of CDC20-associated genes (WebGestalt).

Here, cBioPortal and Cytoscape were enrolled to identify the network of 134 CDC20-associated genes in HCC patients (Supplementary Table 4). Among them, CYP3A4, CYP2E1, CYP2C9, and UGT2B15 were the top 4 genes that had the strongest correlation with CDC20 (Figure 4A). Then we presented the functional enrichment analysis of CDC20 in patients with HCC. Gene Ontology (GO) annotation revealed that CDC20 mainly involved the biological processes such as metabolic process, biological regulation, response to stimulus, and multicellular organismal process. Moreover, cell membrane, endomembrane system, nucleus, and membrane-enclosed lumen were the top 4 cellular components that were associated with the location and function of CDC20. Then the molecular functions correlated with CDC20 were evaluated. Consequently, protein binding, ion binding, hydrolase activity, and nucleic acid binding were on the list (Figure 4B). In addition, the Kyoto Encyclopedia of Genes and Genomes (KEGG) pathway analysis filtered 5 signaling pathways which were the most relevant to CDC20. They were the omega-hydroxylase P450 pathway, epoxygenase P450 pathway, long-chain fatty acid metabolic process, xenobiotic metabolic process, and steroid metabolic process (Figure 4C).

### Correlation between CDC20 expression and tumor infiltrating immune cells

**Figure 5:**
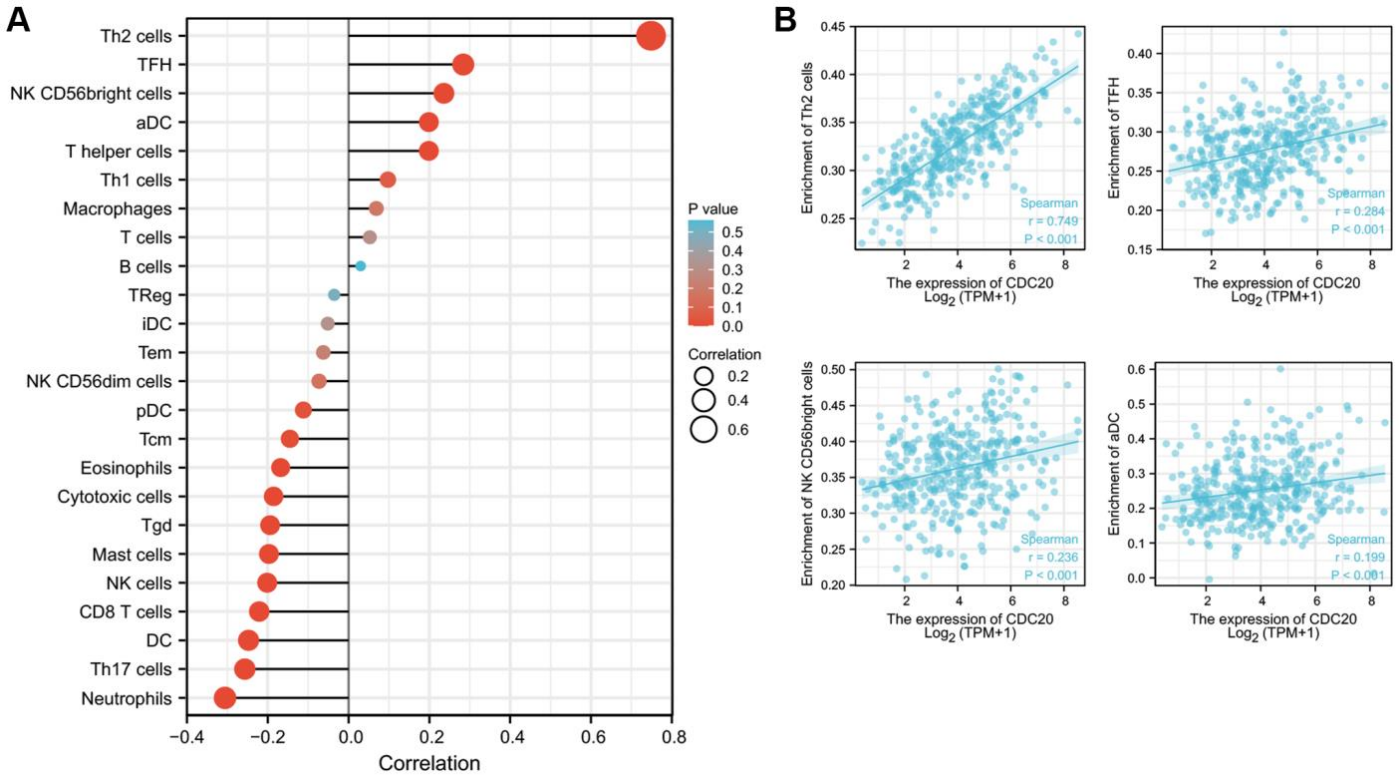
**Relationships between CDC20 expression and tumor-infiltrating immune cells in HCC.** (**A**) Lollipop of Relationships between CDC20 expression and tumor-infiltrating immune cells (XianTao tool). (**B**) The cross-validated association between CDC20 expression and several tumor-infiltrating immune cells, such as T helper 2 cells (Th2), follicular helper T cell (TFH), NK CD56bright cells, and activated dendritic cells (aDC).

We explored the possible correlations between the mRNA expression of CDC20 and levels of immune infiltration in HCC using the XianTao tool. As exhibited in Figure 5, the expression of CDC20 showed significantly positive correlations with the infiltration levels of several different immune cells including T helper 2 cells (Th2), follicular helper T cell (TFH), NK CD56bright cells, and activated dendritic cells (aDC) (Figure 5).

Moreover, we also explored possible correlations between CDC20 expression and other immune signatures by using the TISIDB database. As shown in Supplementary Figure 2A and 2B, CDC20 expression showed significantly positive correlations with the levels of several immunostimulators including TNFRSF18 and MICB. Supplementary Figure 2C and 2D exhibited that CDC20 expression was negatively correlated with several immunoinhibitors including KDR and PDCD1LG2. In addition, the correlations between CDC20 expression and chemokines and chemokine receptors were further assessed. The results showed that CDC20 expression was significantly associated with several chemokines such as XCL1 and CCL26, and several chemokine receptors such as CCR10 and CXCR3 (Supplementary Figure 3). These results indicated that CDC20 could play a critical role in the infiltration and functions of immune cells in HCC.

## DISCUSSION

It is well known that hepatic viruses dramatically contribute to the occurrence and development of HCC through complicated mechanisms which are not fully uncovered. The integration of viral DNA into the host genome incurs the genomic instability, variant mutation, and differential expression of cancer-associated genes [13]. Coinfection of multiple viruses happens commonly in many types of cancer especially in HCC [14–16]. However, little is known about the role of the other viruses (like HTLV-1) in the progression of HCC, as well as their interaction with HBV/HCV. Recently a published study observed a large amount of HCC patients had the coinfection of HTLV-1 and HCV and found that the HTLV-1 infection was independently associated with HCC [12]. These finding suggested the molecular mechanisms underneath was valuable for a better understanding of the initiation, development, and outcome of HCC. Therefore, this present study acquired the list of the HTLV-1 associated genes and co-DEGs in HCC and then identified MAD2L1, CCNB2, CDC20, PTTG1, BUB1B, FOS, and EGR1, as the seven HTLV-1 infection-related genes which were significantly and differentially expressed in HCC tissues through Venn diagram screening.

The following assessments of prognostic value demonstrated that only CDC20 passed this turn of screening, whose high expression was considered to be associated with shorter OS and DSS in HCC. This consequence was in accordance with the notably high expression of CDC20 we found in HCC tissues and cell lines, making CDC20 a potential HTLV-1 infection-associated biomarker and prognostic target for the HCC patients.

Our findings shared novel insights into the association between CDC20 and infection-related HCC on the bases of various studies about CDC20 and cancers. CDC20 is a classic activator of APC, as well as a key E3 ligase with the function of regulating cell cycle checkpoints. The WD40 repeat domain on the protein structure of CDC20 could bind to APC and thereby recognized the substrates such as D-box and KEN box for further proteasomal degradation [17]. The aberrant expression or dysfunction of CDC20 during the metaphase and anaphase of the cell cycle resulted in the abolishment of mitotic arrest, leading to aneuploidy that accounted for carcinogenesis [18]. CDC20 was reported in large amounts to exert its oncogenic role in different types of cancers. For instance, CDC20 promoted the self-renewal of prostate cancer stem-like cells via β-catenin trans-activation, thereby driving the prostate cancer progression [19]. Moreover, there were pieces of evidence indicating that CDC20 accounted for the promotion of radio-resistance of bladder cancer by inducing the degradation of FoxO1 [20]. In addition, the downregulation of CDC20 was reported to increase the activity of the curcumin-mediated anti-tumor treatment against pancreatic cancer [21]. There was also evidence that supported the oncogenic role of CDC20 in glioblastoma via regulating SOX-2-dependent transcription [22]. Our study for the first time explored the oncogenic role of CDC20 in HTLV-1 infection associated HCC.

CDC20 was then chosen for further exploration of its role in biological function, associated signal pathways, and immunoregulation. Immune cells infiltration reflects the efficacy and characteristics of the immune response against the tumor, thus becoming a hotspot these days for intensive study [23, 24]. The combination of the very hopeful immune checkpoint inhibitor therapy and the classic anti-tumor therapies was the future trend of clinical treatment. Here, CDC20 was proved to have a positive correlation with the immune cells including T helper 2 cells, follicular helper T cells, NK cells, and activated dendritic cells. In addition, CDC20 showed a significantly correlation with immunostimulators (TNFRSF18 and MICB), immunoinhibitors (KDR and PDCD1LG2), chemokines (XCL1 and CCL26), and chemokine receptors (CCR10 and CXCR3). T helper 2 cells were recognized to play a pivotal role in mediating anti-tumor immunity. Similar to our finding of the strong association between CDC20 and T helper 2 cells in HTLV-1 infected HCC, a published paper also stated that infection of human herpesvirus 8 induced the T helper 2 immune response which contributed to the formation and proliferation of prostate cancer [25]. TNFRSF18 was served as an immunostimulator that was overexpressed in head and neck squamous cell carcinoma [26]. TNFRSF18 was also observed to be dominated infiltrated by TNFRSF18+ T cells. Besides, the chemokine receptor CCR10 was shown to be involved in the formation and development of inflammation-driven HCC through the activation of PI3K/AKT signaling pathway [27]. The above findings suggested further investigation alongside the clues among CDC20, TNFRSF18, CCR10, and T helper 2 cells might be of great value for HCC patients with HTLV-1 infection.

There are still some limitations in this study. Most results in this research were mainly based on bioinformatics analysis, more experimental verification and molecular mechanism exploration were needed, which will be further improved in subsequent studies.

## CONCLUSIONS

In summary, this study for the first time delineated the correlation of CDC20 with HTLV-1 infection-associated HCC. The disorder of expression and function of CDC20 makes it a probable biomarker for better etiological classification, prognostic prediction, and precision medicine.

## MATERIALS AND METHODS

### Use of bioinformatics tools for acquiring data and reanalysis

The bioinformatics tools and public databases used in this study was summarized in Supplementary Table 5. Briefly, four GSE datasets were obtained from the gene expression omnibus (GEO) database for the identification of co-differentially expressed genes (co-DEGs) in HCC. GSE101685 included 10 normal liver tissues and 24 HCC tissues. GSE112790 included 15 normal liver tissues and 183 HCC tissues. GSE45267 included 39 normal liver tissues and 48 HCC tissues. GSE84402 included 14 paired normal liver and HCC tissues. HTLV-1 infection associated genes were downloaded as a dataset from the MalaCards database. A Venn diagram was used for filtering overlapping genes among the aforementioned datasets.

The prognostic role of CDC20 in HCC was measured via Kaplan–Meier plotter, a public online resource containing information on gene expressions and survival in all kinds of cancers [28–30]. Overall survival and disease specific survival were enrolled here as the two primary endpoints for prognostic analysis. Cox regression was used to perform the multivariate analysis to evaluate the independent risk factors of OS in HCC patients by the XianTao tool.

TNM plot [31] and THPA databases [32] were employed to exhibit the mRNA and protein levels of CDC20 in patients with HCC. A cutoff with statistical significance was set at the point that the *p*-value equals to 0.05. The UALCAN database and XianTao tool was used to collect the clinicopathological features (tumor grades and cancer stages) and CDC expression distribution for correlation analysis [33].

The network of co-expression genes of CDC20 was generated by the cBioPortal database [34], then WebGestalt was recruited to fulfill the functional enrichment analysis including Gene Ontology (GO) analysis and the Kyoto Encyclopedia of Genes and Genomes (KEGG) analysis [35].

TISIDB database, an integrated portal for tumor-immune analysis, was used in this study to illustrate the correlation between CDC20 and immunomodulators [36]. The XianTao tool was used to explore the correlation between CDC20 and immune cells.

### Cells and reagents

Normal human hepatocyte cell line L02 (HL7702), human hepatocellular carcinoma cell lines HepG2 and SK-Hep1 were purchased from the Cell Bank of the Chinese Academy of Sciences (Shanghai, China). Cells were cultured under the normal condition with the 5% CO_2_.

### qPCR and Western blot

qPCR and Western blot were performed following the protocol we previously described [37]. The primer of CDC20 was as followed: F: 5′-ACGGTTTTGATGTAGAGGAAGC-3′; R: 5′-GATACGGTCTGGCAGGGAAG-3′. The primary antibodies of CDC20 (10252-1-AP, Proteintech) and β-tubulin (2146, Cell Signaling Technology) were applied.

### Statistical analysis

Statistical analyses were performed with SPSS 18.0 software (IBM Analytics, USA). All experiments were performed triplicate for statistical analysis, with mean ± SD. Kaplan-Meier analysis was performed to analyze survival rates. The differential mRNA expression between HCC and hepatic cell lines were analyzed using Student’s *t*-test. Cox regression analysis was used for multivariate survival analysis. *p* < 0.05 were defined as statistically significant. ^*^ means *p* < 0.05; ^**^ means *p* < 0.01; ^***^ means *p* < 0.001.

## Supplementary Materials

Supplementary Figures

Supplementary Table 1

Supplementary Tables 2, 3 and 5

Supplementary Table 4
