# Supplementary material for: The bioinformatics and experimental analysis of the novel roles of virus infection-associated gene CDC20 for prognosis and immune infiltration in hepatocellular carcinoma
Source: Aging (Albany NY). 2022 May 27;14(10):4513–29. doi: 10.18632/aging.204093 (PMC9186757; doi:10.18632/aging.204093)
Supplement: Supplementary Figures [file aging-14-204093-s001.pdf]

SUPPLEMENTARY FIGURES

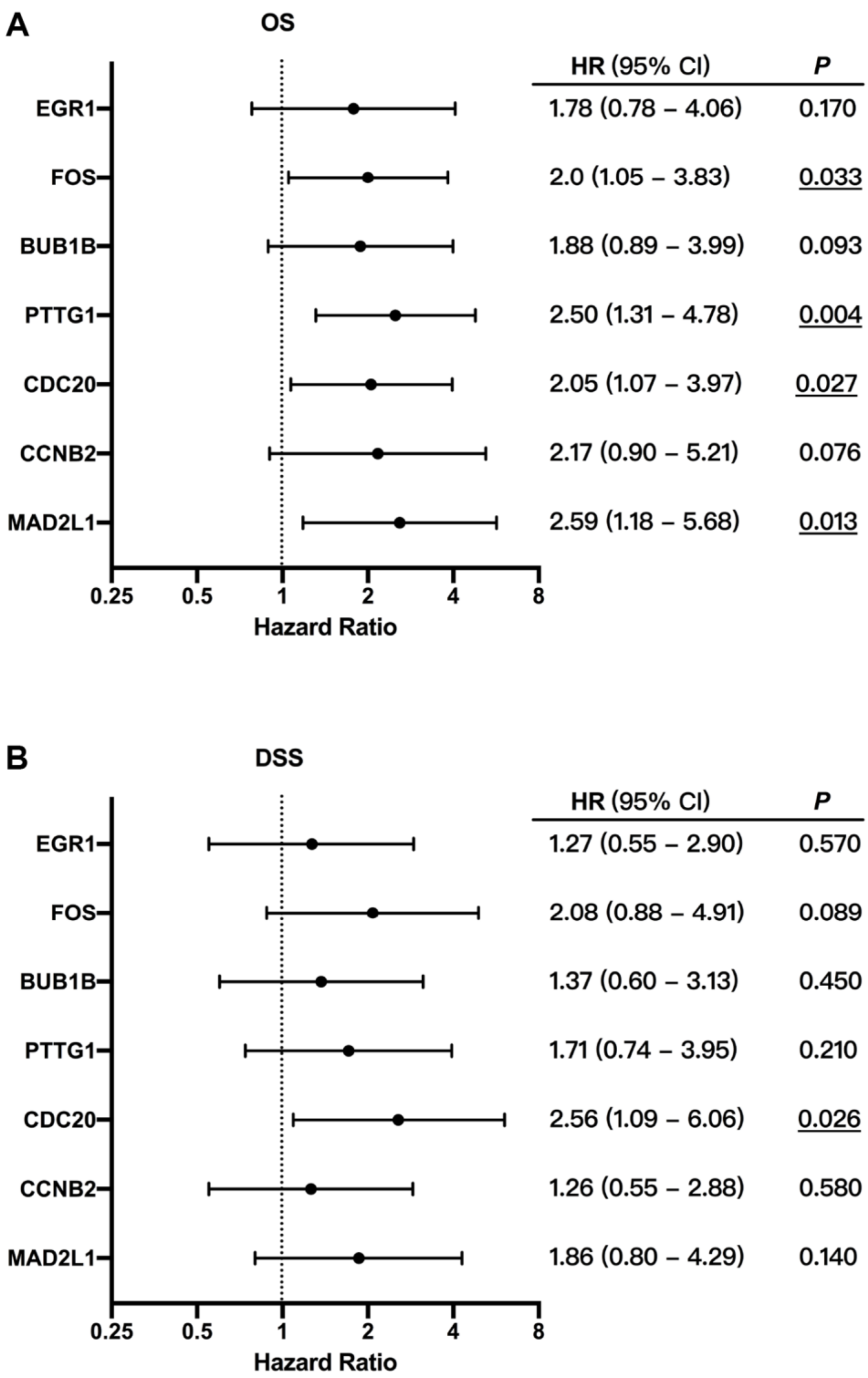

Supplementary Figure 1. (**A** and **B**) Forest plots exhibited the Kaplan-Meier analysis of Overall Survival (OS) and Disease Specific Survival (DSS) in patients with differential expression of each HTLV-1 associated gene.

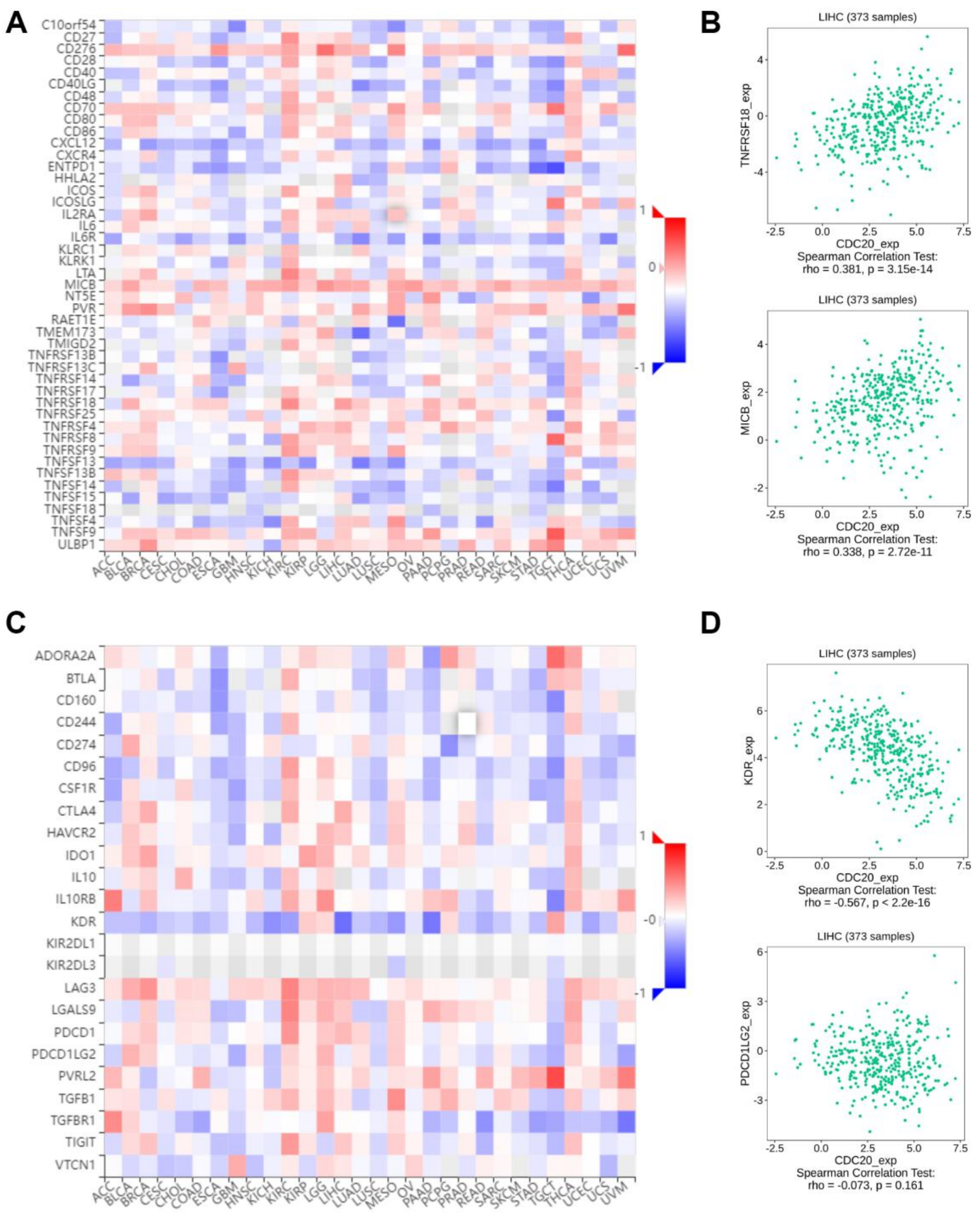

Supplementary Figure 2. The correlation of CDC20 expression with the immunomodulators in HCC. (**A**) The correlation between CDC20 expression and the immunostimulators in pan-cancer including hepatocellular carcinoma. (**B**) The top two immunostimulators with the highest relevance to CDC20. (**C**) The correlation between CDC20 expression and the immunoinhibitors in pan-cancer including hepatocellular carcinoma. (**D**) The top two immunoinhibitors with the highest relevance to CDC20.

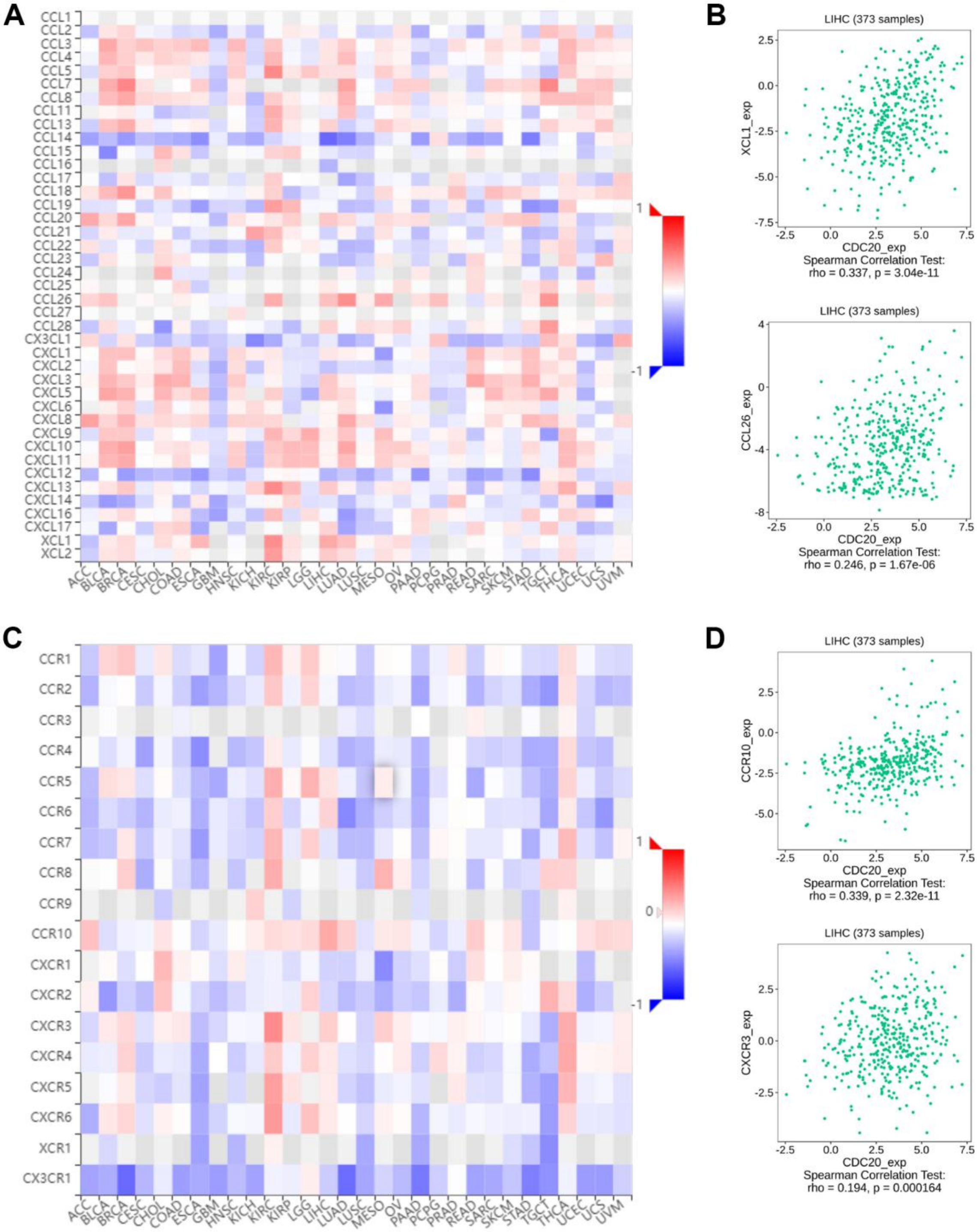

Supplementary Figure 3. The correlation of CDC20 expression with the chemokines and chemokine receptors in HCC. (**A**) The correlation between CDC20 expression and the chemokines in pan-cancer including hepatocellular carcinoma. (**B**) The top two chemokines with the highest relevance to CDC20. (**C**) The correlation between CDC20 expression and the chemokine receptors in pan-cancer including hepatocellular carcinoma. (**D**) The top two chemokine receptors with the highest relevance to CDC20.
