# Supplementary material for: The bioinformatics and experimental analysis of the novel roles of virus infection-associated gene CDC20 for prognosis and immune infiltration in hepatocellular carcinoma
Source: Aging (Albany NY). 2022 May 27;14(10):4513–29. doi: 10.18632/aging.204093 (PMC9186757; doi:10.18632/aging.204093)
Supplement: Supplementary Tables 2, 3 and 5 [file aging-14-204093-s003.pdf]

SUPPLEMENTARY TABLES

Supplementary Table 2. The Genes in the Human T-cell leukemia virus 1 infection.

AKT3	CDK2	CDK4	CDKN1A	CDKN2A	CDKN2B	CDKN2C	ANAPC10	CREB3	KAT5
ADCY2	ADCY3	ADCY5	CHEK1	ADCY6	CHEK2	ADCY7	ADCY8	CHUK	ADCY9
ATF2	CREBBP	ATF6B	CSF2	CREB3L4	DLG1	E2F1	E2F2	E2F3	EGR1
CRTC2	ELK1	ELK4	EP300	AKT1	AKT2	ETS1	ETS2	FDPS	CRTC1
KAT2A	GPS2	SLC25A4	SLC25A5	SLC25A6	ANAPC2	ANAPC4	HLA-A	HLA-B	HLA-C
HLA-DOA	HLA-DOB	HLA-DPA1	HLA-DPB1	HLA-DQA1	HLA-DQA2	HLA-DQB1	HLA-DRA	HLA-DRB1	HLA-DRB3
HLA-E	HLA-F	HLA-G	HRAS	XIAP	ICAM1	IKBKB	IL1R1	IL2	IL2RA
IL6	IL15	IL15RA	ITGAL	ITGB2	JAK1	JAK3	JUN	KRAS	TBPL2
LTBR	MAD2L1	SMAD2	SMAD3	SMAD4	MAP3K1	MAP3K3	MMP7	MSX1	MSX2
ATM	NFATC1	NFATC2	NFATC3	NFATC4	NFKB1	NFKB2	NFKBIA	NFYB	NRAS
ANAPC11	PIK3CA	PIK3CB	PIK3CD	PIK3R1	PIK3R2	POLB	ATR	PPP3CA	PPP3CB
PPP3R2	PRKACA	PRKACB	PRKACG	VAC14	MAPK1	MAPK3	MAPK8	MAPK9	MAPK10
B2M	PTEN	BAX	RAN	RANBP1	RB1	CCND1	RELA	RELB	BCL2L1
CREB3L2	CRTC3	SLC2A1	SPI1	SRF	STAT5A	STAT5B	TBP	TCF3	BUB1B
TGFB2	TGFB3	TGFBR1	TGFBR2	TSPO	TLN1	TNF	TNFRSF1A	TP53	VDAC1
XPO1	ZFP36	IL1R2	FOSL1	CALR	CANX	TRRAP	SLC25A31	TLN2	MAD1L1
PIK3R3	IKBKG	CDC23	NRP1	KAT2B	CDC16	CCNA2	CCNA1	CCND2	CCND3
CREB3L1	CCNB2	CCNE2	CD3D	CD3E	CD3G	BUB3	CD4	PTTG1	TBPL1
ESPL1	CDC20	CDC27	MAP2K2	ANAPC1	TGFB1	VDAC3	RANBP3	MAP3K14	CREB5
ADCY1	TNFRSF13C	EGR2	FOS	HLA-DMA	HLA-DRB4	IL2RB	LCK	MYC	ANAPC5
PPP3CC	MAP2K1	MAP2K4	TERT	VDAC2	CREB3L3	CCNE1	CD40	PPP3R1	ANAPC7
PTTG2	CREB1	ADCY4	CDC26	HLA-DMB	HLA-DRB5	IL2RG	LTA	ATF4	

Supplementary Table 3. Association between multivariable characteristics and overall survival (OS) in HCC using Cox regression.

Characteristics	Total (*N*)	Multivariate analysis	
		Hazard ratio (95% CI)	*P* value
Age	373	1.446 (0.883–2.368)	0.143
≤60	177		
>60	196		
Gender	373	1.007 (0.605–1.675)	0.979
Female	121		
Male	252		
Histologic grade	368	0.894 (0.546–1.462)	0.655
G1&G2	233		
G3&G4	135		
Pathologic stage	349	0.392 (0.022–7.023)	0.525
I&II	259		
III&IV	90		
T stage	370	6.336 (0.366-109.794)	0.205
T1&T2	277		
T3&T4	93		
N stage	258	3.023 (0.398–22.970)	0.285
N0	254		
N1	4		
M stage	272	2.890 (0.810–10.310)	0.102
M0	268		
M1	4		
CDC20	373	1.452 (1.238–1.703)	**<0.001**

Supplementary Table 5. A series of bioinformatics databases for analyzing the novel roles of virus infection-associated gene CDC20 in hepatocellular carcinoma.

Databases	Authors	Samples	Homepage links
TNMplot	Bartha Á, et al.	Tissues	https://tnmplot.com/
The Human Protein Atlas	Anna Asplund, et al.	Tissues	https://www.proteinatlas.org/
UALCAN	Chandrashekar DS	Tissues	http://ualcan.path.uab.edu/index.html
Kaplan-Meier plotter	Gyorffy B, et al.	Tissues	http://kmplot.com/analysis/
cBioPortal	Cerami E, et al.	Tissues	http://www.cbioportal.org/
WebGestalt	Liao Y, et al.	–	http://webgestalt.org/
Cytoscape	Doncheva NT, et al.	–	–
TISIDB	Ru B, et al.	Tissues	http://cis.hku.hk/TISIDB
